# Insights in Anaphylaxis and Clonal Mast Cell Disorders

**DOI:** 10.3389/fimmu.2017.00792

**Published:** 2017-07-10

**Authors:** David González-de-Olano, Iván Álvarez-Twose

**Affiliations:** ^1^Allergy Department, Hospital Universitario Ramón y Cajal, Madrid, Spain; ^2^Instituto de Estudios de Mastocitosis de Castilla La Mancha (CLMast), Hospital Virgen del Valle, Toledo, Spain

**Keywords:** anaphylaxis, clonal, mast cell, mast cell activation syndrome, mastocytosis

## Abstract

The prevalence of anaphylaxis among patients with clonal mast cell disorders (MCD) is clearly higher comparing to the general population. Due to a lower frequency of symptoms outside of acute episodes, clonal MCD in the absence of skin lesions might sometimes be difficult to identify which may lead to underdiagnosis, and anaphylaxis is commonly the presenting symptom in these patients. Although the release of mast cell (MC) mediators upon MC activation might present with a wide variety of symptoms, particular clinical features typically characterize MC mediator release episodes in patients with clonal MCD without skin involvement. Final diagnosis requires a bone marrow study, and it is recommended that this should be done in reference centers. In this article, we address the main triggers for anaphylaxis, risk factors, clinical presentation, diagnosis, and management of patients with MC activation syndromes (MCASs), with special emphasis on clonal MCAS [systemic mastocytosis and mono(clonal) MC activations syndromes].

## Introduction

Anaphylaxis occurs as a result of the sudden release of a wide broad of mediators from mast cells (MCs) and basophils. Clinically, it may show heterogeneous symptoms involving different organs and tissues as far as it fulfils the proposed diagnostic criteria (1), but it usually presents as a serious reaction which can be life threatening or fatal (2).

Mast cells are ubiquous immune cells that preferentially reside as mature cells in the connective tissue from body sites acting as natural barriers for exogenous antigens such as the skin and the gastrointestinal or respiratory tracts, among other tissues; despite this, mature MCs derive from a hematopoietic precursor in the bone marrow (BM). The activation of MCs can be mediated by immunological or non-immunological mechanisms that induce the release of preformed proinflammatory substances and also promote the synthesis of many other mediators (3, 4).

The term MC activation syndrome (MCAS) encompasses a heterogeneous group of disorders characterized by the existence of clinical symptoms secondary to the systemic effects of mediators released by MCs upon their activation, including anaphylaxis. Based on a recent consensus proposal (5, 6), MCAS can be classified into three main categories: (1) primary MCAS, which includes systemic mastocytosis (SM) and (mono)clonal MCAS (MMAS), (2) secondary MCAS, and (3) idiopathic MCAS. The key feature that defines primary MCAS is the demonstration of clonal BM MCs, which results into a constitutive hyperactivity of MCs. In most SM and MMAS patients, MC clonality can be established by the detection of activating mutations of the KIT receptor, a protein membrane involved in the regulation of crucial MC functions such as differentiation, activation and survival. On the contrary, MCs in patients with secondary and idiopathic MCAS are normal; in these latter cases, MC activation symptoms are related with clinical conditions that can secondarily activate MCs such as allergic, neoplastic, inflammatory, or autoimmnune diseases, or with unknown factors.

Herein, we review the main triggers, risk factors, clinical presentation, diagnosis, and management of patients with MCAS, with special emphasis on primary (clonal) MCAS (SM and MMAS).

## Pathophysiology of Anaphylaxis and Clonal MCAS

In allergic reactions, MC activation is due to the interaction of circulating IgE antibodies–antigen complexes with high-affinity Fc receptors for IgE (FcεRI) on the surface of MCs (and basophils). In addition to this mechanism, MCs can also be activated by other non-IgE-mediated immunological mechanisms and by non-immunological mechanisms, such as C3 and C5 (7), nerve growth factor (8), IgG (9–12), and toll-like receptors (13–15), among others. Upon MC activation, the proinflammatory response is further regulated by the balance of both positive and negative multiple molecular events (16), including gp49B1-αvβ3 (17), ITIM and ITAM motifs, kinases, phosphatases, adaptors, and lipids–lipases pathways (16). In parallel, normal and reactive MCs, as well as clonal MCs from patients with primary MCAS, systematically express the stem cell factor receptor (c-kit or CD117) (18), which plays a key role in the regulation of several processes that are crucial for MC function. Similarly to SM patients, the presence of activating *KIT* mutations in clonal MCAS results into a constitutive, ligand-independent hyperactivation of the KIT receptor; this eventually induces the activation of several intracellular downstream signaling pathways involved in differentiation, maturation, migration, activation, and survival of MCs, such as the Ras, Jak, and phosphatidylinositol 3-kinase (PI3K) pathways (17).

## SM and Monoclonal MCASs

Mastocytosis is a heterogeneous group of disorders characterized by the presence of abnormal expansion of clonal MCs in organs and tissues (19, 20). The most recent version (2016) of the World Health Organization classification recognizes several categories of mastocytosis that can be grouped into three main categories of the disease: cutaneous mastocytosis, SM, and MC sarcoma (21). Aditionally, SM can be divided into different subtypes depending on the extent of BM involvement, the existence of signs or symptoms due to end-organ dysfunctions and the presence vs. absence of associated hematologic neoplasms. The most frequent subtype of SM (~80% of all SM cases) is indolent systemic mastocytosis (ISM) (22), which can present with or without skin lesions (ISMs+ and ISMs−, respectively). It is widely accepted that the demonstration of typical skin lesions of mastocytosis in adults leads to the suspicion of SM, and such finding usually initiates the diagnostic work-up of the disease, including a BM evaluation. By contrast, ISMs− (~20% of all ISM cases) is frequently underdiagnosed, mainly due to the heterogeneity and the lack of specificity of presenting clinical symptoms that can overlap with those found in more common allergic diseases (23). In this regard, the demonstration of increased levels of serum baseline tryptase (sBT), a protease which is almost exclusively released by MCs, has contributed for a better identification of ISMs− cases; despite this, a subset of patients with ISMs−, particularly those who have a low BM MC burden, may show low (even normal) sBT levels. Altogether, these findings support the need for additional (prediagnostic) criteria that could help to determine the risk of having an underlying clonal MCAS in patients suffering from MC mediator release symptoms, in order to properly select potential candidates for a BM study (24).

In recent years, the term MCAS has emerged to encompass all those clinical entities characterized by MC activation, including SM. In general terms, MCAS is defined by (i) the presence of recurrent signs or symptoms attributable to the release of MC mediators, together with (ii) increased levels of biochemical markers of MC degranulation in blood and/or urine, and (iii) response to MC stabilizers and/or MC mediator-targeted drugs (6). The European Competence Network on Mastocytosis (ECNM) has recently proposed a comprehensive classification of MCAS (25), in which three main categories of MCAS are recognized depending on whether the cause of MC activation is the presence of a clonal expansion of MCs (primary MCAS), the existence of disorders that can potentially induce MC degranulation such as allergy, inflammatory, and autoimmune diseases or tumors (secondary MCAS), or unknown (idiopathic MCAS) (5, 6). As some patients with primary MCAS (e.g., SM) can also present with secondary causes of MC activation (e.g., allergy) or fulfill diagnostic criteria for idiopathic entities of MCAS (i.e., idiopathic anaphylaxis), the Spanish Network on Mastocytosis (REMA) has proposed to classify MCAS in only two main groups (i.e., clonal and non-clonal MCAS) based on the presence vs. absence of clonal BM MCs, respectively. In any case, a complete BM evaluation should be necessary in all patients with suspected MCAS in order to discriminate between entities presenting with clonal (primary) MCAS, including SM and (mono)clonal MCAS (MMAS), and non-clonal (secondary and idiopathic) MCAS. Despite this, non-clonal MCAS are frequently assumed in clinical practice in the absence of BM evaluation; in turn, primary MCAS may represent a diagnostic challenge due to the lack of specificity of their clinical symptoms and the need of highly sensitive diagnostic techniques to establish the clonal nature of MCs, as discussed in detail below.

From a pathogenic point of view, the most relevant biological finding in SM (and also in MMAS) is the presence of activating *KIT* mutations (mostly the Asp816Val -D816V- *KIT* mutation) in the vast majority of cases (26–29), which results into a constitutive, ligand-independent, activation of the KIT receptor. In virtually all patients with SM, the existence of activating *KIT* mutations is accompanied by the aberrant expression of CD25 (and/or CD2) on BM MCs, which is therefore widely considered as a surrogate marker of MC clonality (30). Both genetic and immunophenotypic features suggest a profound alteration in the mechanisms of adhesion, activation and migration of MCs (31). Despite MC mediator release symptoms, MC clonality and increased sBT levels are findings commonly shared by SM and MMAS, the distinction between both entities can be established by the absence of enough criteria for the diagnosis of SM in MMAS patients, as further discussed herein.

## Epidemiology, Triggers, and Risk Factors of Anaphylaxis in Clonal MCAS

Different allergic diseases such as rhinitis, conjunctivitis, asthma, urticaria, and atopic dermatitis have been reported to be present in patients with mastocytosis, with a similar frequency as that found in the general population (32). Nevertheless, the prevalence of anaphylaxis has been reported to be up to 100 times more frequent among patients with SM vs. general population (33, 34) with an overall frequency that ranges from 22 and 49% in adults (32, 35, 36) and between 6 and 9% in children (32, 35). Furthermore, it seems to occur more often in patients with clonal MC disease without cutaneous involvement (24, 37). In fact, anaphylaxis is commonly the presenting symptom in patients with ISMs− and MMAS (18, 34, 38). Although the existence of a clonal MCAS is a predisposing factor for severe MC mediator release episodes by itself, other factors have been associated with an increased risk for the development or the severity of anaphylaxis among patients with SM and MMAS (35). In adults with SM, it has been suggested that the overall BM MC burden is inversely related with the severity of MC mediator release symptoms. Thus, ISM patients (particularly those with ISMs−) suffer from anaphylaxis more frequently than patients with advanced forms of SM (i.e., aggressive SM) (24, 39).

**Figure 1:**
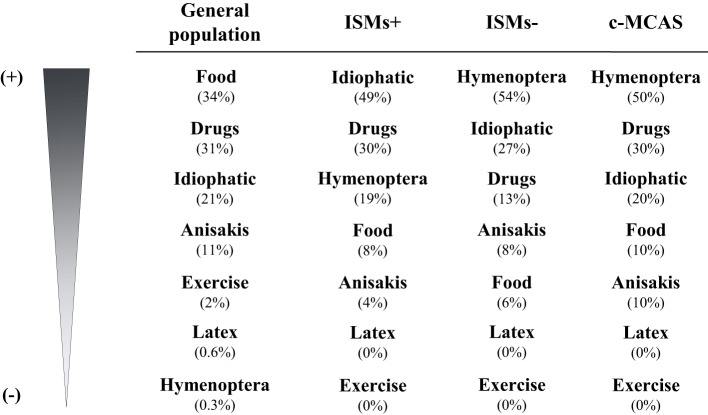
Main triggers of anaphylaxis in general population vs. clonal MCAS patients. Modified from Ref. (18). ISMs+, indolent systemic mastocytosis with skin lesions; ISMs−, indolent systemic mastocytosis without skin lesions; c-MCAS, (mono)clonal mast cell activation syndromes.

A wide variety of elicitors such as insects, drugs, food, as well as physical, environmental, and emotional factors have been recurrently reported as potential triggers of MC activation episodes in patients with MCAS (18, 24, 40). Among these, the most common trigger of anaphylaxis in adults with ISMs− and MMAS is, by far, hymenoptera sting, followed by unknown cause (idiopathic) and drugs (Figure 1). A recent study by the REMA (38) has suggested that ISMs− associated with anaphylaxis exclusively triggered by insects (mostly hymenoptera) represents a subtype of ISMs− that rarely refer anaphylaxis with additional elicitors (38, 40) and displays unique clinical, biological, and molecular features vs. ISMs− triggered by other factors and ISMs+. These features include (1) a clear male predominance, (2) a typical clinical profile of acute MC mediator release episodes characterized by (cardio)vascular symptoms in the absence of urticaria and angioedema, (3) a low BM MC burden, and (4) the detection of the *KIT* mutation restricted to the MC compartment. Importantly, the extent of involvement of hematopoiesis by the *KIT* mutation—restricted to MC or affecting additional hematopoietic cell lineages—has been reported to be the most relevant risk factor for disease progression in ISMs−; for this reason, ISMs− associated with insect-induced anaphylaxis appears to be the subtype of ISM with more favorable long-term prognosis (41).

## Clinical Presentation of Anaphylaxis in Clonal MCAS

Upon MC activation, a wide variety of symptoms can occur as a result of the systemic effect of proinflammatory and vasoactive mediators released from MCs, which ranges from pruritus, hives, flushing, tachycardia, abdominal pain, or diarrhea, to syncopal or near-syncopal episodes. Several studies by the REMA have shown that MC mediator release episodes in patients with ISMs− (and MMAS) are typically characterized by cardiovascular symptoms (i.e., dizziness and/or syncope) without cutaneo-mucosal symptoms (i.e., urticaria and angioedema) (24). These observations, together with a male predominance of ISMs− and increased levels of sBT, led to the development of a predictive model (REMA score) (Table 1), which showed a high efficiency to discriminate between patients with clonal MCAS and other types of MCAS (24, 42). Interestingly, the REMA score also showed higher specificity and sensitivity compared to the evaluation of sBT levels alone for predicting SM and MMAS. Based on these results, the ECNM has incorporated the REMA score in the most recent consensus algorithm for the diagnosis of SM (25). Moreover, the systematic application of the REMA score in patients presenting with symptoms related with MC activation in the absence of skin lesions evaluated by the REMA has allowed not only to decrease the number of BM studies carried out in these group of patients, but also to identify an increasing number of patients with ISMs− (and MMAS) showing normal sBT levels (~10% of all ISMs− cases in the updated series of the REMA, data not published). These observations, together with the simplicity and the low cost of the method to select for potential candidates for BM evaluation, highlights the clinical and socioeconomic impact of the REMA score and supports its application in a routine basis in clinical practice.

**Table 1:** REMA score proposed to predict clonal MCAS in patients in the absence of skin lesions.

Variable	Score
**Gender**	
Male	+1
Female	−1
**Clinical symptoms**	
Absence of urticaria, pruritus, and angioedema	+1
Urticaria, pruritus, and/or angioedema	−2
Presyncope and/or syncope	+3
**Baseline serum tryptase**	
<15 ng/ml	−1
>25 ng/ml	+2

*Reprinted from Álvarez-Twose et al. (42). Reproduced with permission of Karger*.

*MCAS, mast cell activation syndrome*.

*Score <2: low probability of clonality*.

*Score ≥2: high probability of clonality*.

## Diagnosis of Clonal MCAS

The distinction between primary MCAS (ISMs− and MMAS) and secondary or idiopathic MCAS is based on the demonstration on the clonal nature of MCAS. Given the fact that MCs are produced in the BM and that *KIT* mutation is usually restricted to the MC compartment (24), the establishment of MC clonality requires the study of the BM.

The diagnosis of SM is based on the coexistence of one major criterion and one minor criterion, or ≥3 minor criteria in the absence of the major criterion (21). The major criterion consists on the demonstration of multifocal aggregates of ≥15 MCs in BM sections (or in other extra-cutaneous tissues). In turn, the minor criteria include (i) identification of >25% of morphologically abnormal MCs in BM smears, (ii) demonstration of an aberrant expression of CD25 and/or CD2 on MCs, (iii) detection of the activating mutations in codon 816 of the *KIT* gene, and (iv) presence of sBT levels ≥20 μg/l. In contrast to SM, the diagnosis of MMAS is established when only one or two minor criteria (not including increased sBT levels) are present in the absence of the major criterion (43–45).

Whereas *KIT* mutations and the aberrant expression of CD25 on BM MCs can be already detected in early stages of SM as long as highly sensitive techniques are applied (46), the presence of BM MC aggregates and increased levels of sBT are closely related with the proliferation rate of the clonal MC population in SM and can be absent in a significant proportion of patients with ISMs−. Given the low (frequently very low) BM MC burden that characterizes both ISMs− and MMAS, it must be emphasized that BM studies in patients with symptoms related with MC activation without skin lesions should include highly sensitive diagnostic techniques in order to detect clonal MCs even when they represent only a minority of the nucleated cells in the BM (24). Among these techniques, multiparametric flow cytometry and molecular methods on fluorescence-activated cell sorting-purified BM MCs such as peptide nucleic acid-mediated polymerase chain reaction (PCR) clamping or allele-specific oligonucleotide quantitative PCR, are preferred over other methods (i.e., CD25 immunohistochemistry and conventional PCR) to establish the clonal nature of BM MCs in this clinical setting (47). Accordingly, it is strongly recommended to perform the BM study of patients with suspected SM (particularly those without skin involvement) in highly specialized reference centers for mastocytosis.

## Treatment of Anaphylaxis in Patients with Clonal MCAS

### Acute Treatment

It is of paramount importance that clinicians early recognize and treat MC mediator release symptoms (48). As among general population, treatment with epinephrine (adrenaline) injected intramuscularly in the mid-outer thigh, as soon as anaphylaxis is diagnosed or strongly suspected, constitutes the first line treatment of anaphylaxis, and repeated doses might be administered after 5–15 min in the absence of optimal response (2). At the same time, life-sustaining treatment including supplemental oxygen or intravenous fluids should be administered as needed (49). H1 antihistamines and H2 antihistamines usually in combination with glucocorticoids are considered as second-line medications in anaphylaxis. In addition, other coadjuvant drugs might be administered depending on the presenting clinical features of the patients.

### Baseline Treatment

Treatment strategies for clonal MCAS do not significantly differ from those used in other well-known entities cursing with secondary or idiopathic MC activation, and they are focused on preventing and/or decreasing the effects of mediators released from MCs. These strategies include an adequate information and training of the patient, their relatives, and care providers in order to avoid triggers (18), and the administration of different anti-mediator therapy selected on the basis of the intensity and/or severity of the signs and symptoms linked with the activation of MCs (46) such as histamine receptors blockers, sodium cromolyn, leukotriene antagonists, corticosteroids, and epinephrine, among others.

H1 blockers have been shown to decrease pruritus, flushing, urticaria, tachycardia, hypotension, and abdominal pain related with MC degranulation (50, 51). H2 antihistamines seem to potentiate the effect of H1 antihistamines and can also be of potential utility in patients with suboptimal response to H1 blockers alone (52). Oral sodium cromolyn is a MC stabilizer, which has proven to decrease symptoms such as diarrhea, abdominal cramping, nausea, pruritus, flushing, bone pain, headache, and some cognitive symptoms in patients with mastocytosis (53). In selected cases, aspirin and COX-2 selective inhibitors such as celecoxib might be also useful whenever previous tolerance to such drugs has been demonstrated (52).

In refractory cases despite conventional therapy, treatment with the anti-IgE recombinant humanized monoclonal antibody omalizumab has shown to suppress MC activation episodes in all clinical subtypes of MCAS presenting as idiopathic anaphylaxis (54–56), Meniere’s disease (57), and also to prevent reactions related to venom immunotherapy administration (58, 59). The mechanism by which omalizumab decreases the release of MC mediators in asthma (60) and in spontaneous chronic urticaria (61) appears to be related with its ability to block the binding of circulating IgE antibodies to FcεRI receptors on the surface of MCs and basophils resulting into a decrease on receptor expression (62), or by interfering with the release of MC mediator (63). Although it has not been confirmed so far, the response to omalizumab reported in a few primary MCAS patients (54–56) may not differ from the mechanisms referred above.

More rarely, some patients with SM showing high BM MC burden could benefit from cytorreductive or immunomodulatory drugs such as hydroxyurea (64), interferon alpha2b (65), and cladribine (2-CDA) (66) among others. More recently, several tyrosine kinase inhibitors (TKIs) such as midostaurin, masitinib, or dasatinib have shown to improve MC mediator release symptoms in a subset of patients with SM, even in the absence of significant decrease in BM MC numbers (52); despite this, the usage of TKI drugs should be restricted to highly symptomatic SM patients unresponsive to conventional intensive anti-mediator therapy, ideally in the setting of clinical trials.

## Concluding Remarks

Emerging entities with clonal BM MCs as ISMs− and clonal MCAS might sometimes be difficult to recognize. Anaphylaxis is commonly the presenting symptom, and hymenoptera sting is the most common trigger. Both entities involve a great challenge either from the diagnostic or therapeutic point of view. Final diagnosis requires a BM study, but given the low BM MC burden typical of these entities, highly sensitive techniques are mandatory.

## Author Contributions

Both authors have contributed to the conception, design, and drafting of the paper.

## Conflict of Interest Statement

The authors declare that the research was conducted in the absence of any commercial or financial relationships that could be construed as a potential conflict of interest.
